# A New Method to Monitor the Contribution of Fast Food Restaurants to the Diets of US Children

**DOI:** 10.1371/journal.pone.0103543

**Published:** 2014-07-25

**Authors:** Colin D. Rehm, Adam Drewnowski

**Affiliations:** 1 Center for Public Health Nutrition, University of Washington, Seattle, Washington, United States of America; 2 Institute for Cardiometabolism and Nutrition, Groupe Hospitalier Pitié-Salpêtrière, Paris, France; Old Dominion University, United States of America

## Abstract

**Background:**

American adults consume 11.3% of total daily calories from foods and beverages from fast food restaurants. The contribution of different types of fast food restaurants to the diets of US children is unknown.

**Objective:**

To estimate the consumption of energy, sodium, added sugars, and solid fats among US children ages 4–19 y by fast food restaurant type.

**Methods:**

Analyses used the first 24-h recall for 12,378 children in the 2003–2010 cycles of the nationally representative National Health and Nutrition Examination Survey (NHANES 2003–2010). NHANES data identify foods by location of origin, including stores and fast food restaurants (FFR). A novel custom algorithm divided FFRs into 8 segments and assigned meals and snacks to each. These included burger, pizza, sandwich, Mexican, Asian, fish, and coffee/snack restaurants. The contribution of each restaurant type to intakes of energy and other dietary constituents was then assessed by age group (4–11 y and 12–19 y) and by race/ethnicity.

**Results:**

Store-bought foods and beverages provided 64.8% of energy, 61.9% of sodium, 68.9% of added sugars, and 60.1% of solid fats. FFRs provided 14.1% of energy, 15.9% of sodium, 10.4% of added sugars and 17.9% of solid fats. Among FFR segments, burger restaurants provided 6.2% of total energy, 5.8% of sodium, 6.2% of added sugars, and 7.6% of solid fats. Less energy was provided by pizza (3.3%), sandwich (1.4%), Mexican (1.3%), and chicken restaurants (1.2%). Non-Hispanic black children obtained a greater proportion of their total energy (7.4%), sodium (7.1%), and solid fats (9.5%) from burger restaurants as compared to non-Hispanic white children (6.0% of energy, 5.5% of sodium, and 7.3% of solid fat).

**Conclusions:**

These novel analyses, based on consumption data by fast food market segment, allow public health stakeholders to better monitor the effectiveness of industry efforts to promote healthier menu options.

## Background

Consumption of foods away from home (FAFH) is thought to contribute to poor diet quality among children and adults [1]–[6]. FAFH represent a broad category that can include meals and snacks eaten at restaurants, schools, or at entertainment or sports events. The impact of fast foods on diet quality of children has received much research attention [7]–[11]. Nutritional analyses of fast food menu offerings have focused on the amount of energy, sodium, added sugars, and fats for fast food meals and menu items [12]–[14].

Since 2003, the National Health and Nutrition Examination Survey (NHANES), the primary source of dietary surveillance data in the US, has coded all foods and beverages consumed by their location of origin, which is reported by survey respondents. Among such locations were supermarkets and grocery stores, fast food restaurants/pizza (FFR), full-service restaurants (FSR), school cafeterias, and vending machines, among many others. The fast food industry generally segments fast food restaurants into 8 different types: burger, pizza, sandwich, chicken, Mexican, Asian, and fish restaurants, as well as coffee/snack shops [15]. However, the NHANES dataset does not distinguish among different FFRs by restaurant type [10], [16].

The present study provides the first analysis of children’s consumption patterns by the type of FFR. Custom algorithms, developed by the authors, subdivided the NHANES FFR/pizza category into 8 different types. Meals and snacks consumed at each type of restaurant were then analyzed for energy, sodium, added sugars, and solid fats. The present focus was on the relative contribution of different types of FFR to US children’s diets.

Analysis of actual consumption data, instead of menu offerings, can provide an alternate picture of the contribution of the fast food industry to the diets of US children and youth. Detailed analyses by FFR type will also allow for better monitoring of food industry trends by interested public health stakeholders. The present study is the first-ever analysis of children’s diets by specific type of fast food restaurant. Rather than assess the caloric contribution of specific food items (e.g. burgers or pizza) [16], [17], the present analyses provide the first estimate of calories, sodium, added sugars and solid fats from burger or pizza restaurants.

## Methods

### Dietary intake data sources

Data analyses were based on the first 24-h recall from 4 cycles of the nationally representative National Health and Nutrition Examination Survey (NHANES) for the years 2003–2010. The NHANES 24-h dietary recall utilizes a multi-pass method, where all foods and beverages consumed in the preceding 24-h, from midnight to midnight were reported. The name, time and place of consumption for each eating occasion were also measured. For children 4–5 y, a parent or guardian completed the dietary recall. For children 6–11 y, the child was the primary respondent, but the parent was present and able to assist. For children 12–19 y, the child was the primary respondent, but could be assisted by an adult [18].

Data from the MyPyramid Equivalents Database (MPED) were used to assess intakes of added sugars and solid fats [19]. The MPED 2.0 database was updated for use with more recent NHANES cycles by imputing the MPED equivalents for a limited number of foods (n = 291). Energy from solid fats was estimated as 9 kcal/g of solid fat. Since the MPED database provided added sugars in teaspoon equivalents, this value was converted to energy (1 tsp = 16 kcal) [20].

The 5 principal locations of origin for all foods and beverages were obtained from NHANES data. These were: grocery stores, fast food restaurants (FFR), full-service restaurants (FSR), school cafeterias, and other [21]. The “other” category included foods or beverages from someone else or as a gift, child-care centers, sports/recreational facilities, vending machines, and grown or caught among other categories [21]. Since no further data about the FFR category are provided in NHANES data, a multi-step algorithm was developed by the authors to assign FFR meals and snacks into one of 8 market segments as defined by the restaurant industry [15].

### The FFR segmentation

The 8 FFR segments were burger restaurants (e.g., McDonald’s or Burger King), pizza restaurants (e.g., Pizza Hut or Domino’s), sandwich restaurants (e.g., Subway or Quiznos), chicken restaurants (e.g., KFC or Chick-fil-A), Mexican restaurants (e.g., Taco Bell), Asian restaurants (e.g., Panda Express), fish restaurants (e.g., Long John Silver’s) and coffee/snack shops (e.g., Starbucks or Baskin-Robbins). The NHANES data included meals and snacks from national chains as well as regional or local restaurants: the specific brand names provided above are for reference only.

FFR meals were assigned to one of the eight pre-defined segments based on the foods and beverages consumed at that meal. First, unique meals were defined as eating occasions that occurred at the same time and at the same location (i.e., at-home or away from home). Both fast foods consumed at-home and away-from-home were included in the coding algorithm; the place of eating was simply used to define unique eating occasions. Second, an iterative algorithm scanned the 24-h recall individual foods file for “sentinel” foods that could be used to identify a specific market segment. Among the 26 sentinel foods were burgers, pizza, Mexican dishes (e.g., tacos or burritos), chicken strips/nuggets, fried chicken, submarine/deli sandwiches, and hot breakfast items as well as pretzels, hot dogs, side-dishes often served with fried chicken (e.g., baked beans, mashed potatoes and gravy, biscuits, potato salad, where no fried chicken was served), French fries alone, or soda alone.

Foods and food combinations (e.g., hamburger, fried chicken, or pizza) indicated the type of fast food restaurant. If a given meal occasion included only one of these sentinel main dishes, it was coded as such. Examples of meal components that were readily coded and assigned to a given FFR segment were burgers, pizza, Mexican dishes, fried chicken (but not chicken strips/nuggets), or Asian dishes (e.g., fried rice or General Tso’s chicken). Meals that contained multiple potential main dishes were flagged for further refinement (described below). Most of the meal components could be unambiguously assigned to a single and identifiable FFR segment. The initial scan coded 77.3% of single items, while the remainder was assessed manually.

For some meals, the assignment to a FFR segment was more complex. For example, chicken nuggets/strips are typically sold at both burger and chicken restaurants. Other examples include soda consumed alone, hot breakfast dishes, French fries alone, or ice cream. These meals were randomly assigned to each segment according to a deterministic probability based on weights from sales data for the 50 largest chains. For example, all chicken and burger establishments selling a chicken nugget/strip product were identified and assigned a weight to each segment that randomly divided such meals according to weighted sales. This approach assumes that the relative revenues from each product were similar across different brand segments. For the chicken strips/nugget meal component, 84.3% were assigned to the burger segment while 15.7% were applied to the chicken segment [15].

The reliability of the algorithm was evaluated by an independent coder who assigned 138 random meals (412 individual food items) into the 8 FFR segments. The chance-corrected concordance (Kappa) was estimated at 0.89, indicating a high level of agreement. Some refinements were made to the algorithm following the reliability sub-study. Specifically, sweet and sour sauce was originally coded as an indicator food for Asian-type dishes, but it became clear it was most often associated with eating chicken nuggets/strips.

### Analytical approach

Analyses were conducted for all children (ages 4–19 y) and by age group (ages 4–11 and 12–19 y). Three race/ethnicity groups were examined: non-Hispanic whites, Mexican-Americans and non-Hispanic blacks. The race/ethnicity analysis excluded a sub-sample of the population for which presentation of race/ethnicity specific estimates is not recommended [22]. The three race/ethnicity groups examined constituted 89% of the population.

Dependent measures were dietary energy (kcal/d), sodium (mg/d), added sugars (kcal/d) and solid fats (kcal/d). These dietary constituents were most frequently cited in past analyses of foods away from home, and our important components of summary measures of diet quality [12]–[14], [23], [24]. Two summary measures were assessed: the survey-weighted mean and the survey-weighted population proportion. The population proportion is the percent of each dietary constituent provided by FFR segment. This measure, interpreted as a ratio of the means, rather than a mean of the ratios, is best suited for examinations of population-level habits [25].

Additional analyses examined the energy-adjusted contribution of these dietary constituents by NHANES location of origin and FFR segment. Sodium was presented in mg/1000 kcal, and energy from added sugars and solid fats as a percentage of total energy from that segment.

A survey-weighted Wald test was used to determine the statistical significance of differences in means and proportions between sub-populations by age and race/ethnicity, with adolescents (12–19 y) and non-Hispanic adults as the reference groups. A total of 12,378 children and adolescents were included in all analyses, with the exception of those by race/ethnicity, which included 10,847. All analyses accounted for the complex NHANES stratified multistage sampling design and for over-sampling. The analysis also accounted for survey non-response and all results were representative of the US child population from 2003–2010. Analyses were conducted using Stata 12.1 (StataCorp, College Station, TX).

### Data availability and ethical approval

The necessary IRB approval for NHANES had been obtained by the National Center for Health Statistics (NCHS) [26]. The study was exempt from human subjects review per University of Washington policies. All data used here are publicly available on the NCHS website [27].

## Results

### Consumption patterns by NHANES location

**Table 1** shows the survey-weighted means and population proportion for energy, sodium, added sugars and solid fats by NHANES food location and age group. Overall, foods and beverages from supermarkets and grocery stores accounted for the bulk of dietary energy (65%), sodium (62%), added sugars (69%) and solid fats (60%). FFRs were the second most important source of these dietary factors, contributing 14% of total energy, 16% of sodium, 10% of added sugars, and 18% of solid fats. FFRs contributed more energy to the diets of 12–19 y olds (16.5%) than to the diets of 4–11 y olds (11.2%). Approximately 21% of total energy came from FSRs, schools, and other sources.

**Table 1:** Mean and % of total for energy and selected dietary factors (and standard errors) by location of origin.

	Energy (kcal)		Sodium (mg)		Added sugars (kcal)		Solid fats (kcal)	
	Mean	% of total	Mean	% of total	Mean	% of total	Mean	% of total
Overall (age 4–19 y)								
Store	1345 (13)	64.8 (0.6)	2005 (25)	61.9 (0.7)	238 (4)	68.9 (0.6)	252 (3)	60.1 (0.7)
Fast food restaurant	293 (9)	14.1 (0.4)	515 (16)	15.9 (0.5)	36 (1)	10.4 (0.4)	75 (2)	17.9 (0.5)
Full-service restaurant	116 (7)	5.6 (0.4)	226 (16)	7.0 (0.5)	16 (1)	4.5 (0.3)	26 (2)	6.2 (0.4)
School	150 (9)	7.2 (0.4)	247 (17)	7.6 (0.5)	17 (1)	4.9 (0.4)	34 (2)	8.1 (0.5)
Other	171 (5)	8.2 (0.3)	248 (9)	7.6 (0.3)	39 (2)	11.4 (0.5)	32 (1)	7.7 (0.4)
Age 4–11 y								
Store	1246 (16)	65.6 (0.8)	1854 (26)	63.9 (0.9)	204 (4)	66.9 (1.0)	245 (5)	62.3 (1.0)
Fast food restaurant	212 (11)	11.2 (0.6)	356 (20)	12.3 (0.7)	27 (2)	8.8 (0.5)	54 (3)	13.8 (0.8)
Full-service restaurant	84 (7)	4.5 (0.4)	158 (14)	5.5 (0.5)	12 (1)	4.1 (0.4)	18 (2)	4.7 (0.5)
School	173 (11)	9.1 (0.6)	277 (19)	9.5 (0.6)	19 (1)	6.3 (0.5)	39 (3)	10.0 (0.7)
Other	184 (8)	9.7 (0.4)	258 (12)	8.9 (3.9)	42 (3)	13.9 (0.8)	36 (2)	9.2 (0.5)
Age 12–19 y								
Store	1441 (19)	64.2 (0.7)	2150 (40)	60.3 (9.5)	271 (6)	70.3 (0.7)	258 (5)	58.2 (0.9)
Fast food restaurant	371 (12)	16.5 (0.5)	668 (24)	18.7 (0.7)	45 (2)	11.6 (0.4)	95 (3)	21.4 (0.7)
Full-service restaurant	147 (10)	6.5 (0.5)	291 (26)	8.2 (0.7)	19 (2)	4.9 (0.4)	33 (3)	7.5 (0.6)
School	127 (11)	5.6 (0.5)	219 (20)	6.1 (0.5)	15 (2)	3.8 (0.4)	29 (3)	6.5 (0.6)
Other	159 (7)	7.1 (0.4)	237 (14)	6.7 (0.4)	36 (2)	9.4 (0.5)	29 (2)	6.5 (0.4)

### Consumption patterns by fast food restaurant type

Overall, 35.7% (95% CI 33.9–37.7%) of all children ages 4–19 y consumed fast food items on a given day. Seventeen percent (95% CI 16.1–18.8%) of all children consumed items from burger restaurants, whereas 9.0% (95% CI 8.0–10.1%) consumed items from pizza restaurants. About 4% of all children consumed any items from sandwich, chicken, and Mexican FFRs.

**Table 2** provides the mean and population proportion of energy by fast food restaurant type and age group. Within the FFR category, burger restaurants provided the most energy (6.2% overall, 5.4% for 4–11 y olds and 6.8% for 12–19 y olds). Among the FFR types, pizza restaurants provided the second most energy (3.3% overall, 2.7% for 4–11 y olds, and 3.9% for 12–19 y olds). Overall, sandwich, chicken and Mexican restaurants each provided less than 1.5% of total energy. The Asian, coffee/snack and fish segments each provided less than 0.5% of total energy. Given the strong correlation between energy and other dietary components, the contribution of each FFR segment to sodium (**Table 3**), added sugars (**Table 4**) and solid fats (**Table 5**) tracked the results for energy. Among the different FFR segments, burger restaurants provided the most sodium, added sugars and solid fats.

**Table 2:** Mean energy and % of total energy (and standard errors) by FFR segment and age group.

	Total (n = 12,378)		Age 4–11 y (n = 5,681)		Age 12–19 y (n = 6,697)	
	Mean	% of total	Mean	% of total	Mean	% of total
Burger	128 (6.1)	6.2 (0.3)	102 (8.1)†	5.4 (0.4)^‡^	154 (6.7)	6.8 (0.3)
Pizza	69 (4.8)	3.3 (0.2)	51 (5.9)†	2.7 (0.3)^‡^	87 (7.3)	3.9 (0.3)
Sandwich	29 (2.2)	1.4 (0.1)	15 (2)†	0.8 (0.1)†	42 (3.9)	1.9 (0.2)
Chicken	26 (2.3)	1.2 (0.1)	23 (3.3)	1.2 (0.2)	28 (3.6)	1.2 (0.2)
Mexican	27 (2.4)	1.3 (0.1)	12 (2.7)†	0.6 (0.1)†	41 (4)	1.8 (0.2)
Asian	9 (1.4)	0.4 (0.1)	4 (1.2)^‡^	0.2 (0.1)^‡^	13 (2.4)	0.6 (0.1)
Coffee/snack	3 (0.5)	0.2 (0.0)	3 (0.6)	0.2 (0.0)	4 (0.8)	0.2 (0.0)
Fish	3 (0.9)	0.1 (0.0)	1 (0.7)	0.1 (0.0)	4 (1.6)	0.2 (0.1)
Other sources	1782 (14.2)	85.9 (0.4)	1688 (13.8)^‡^	88.8 (0.6)†	1873 (21.7)	83.5 (0.5)
Total energy	2076 (13.7)	–	1900 (15.4)†	–	2244 (21.0)	–

† p<0.001; ^‡^0.001<p<0.01 for difference between two age groups. Significance testing conducted separately for mean and % of total energy. Value in parentheses is the standard error.

**Table 3:** Mean sodium and % of total sodium (and standard errors) by FFR segment and age group.

	Total (n = 12,378)		Age 4–11 y (n = 5,681)		Age 12–19 y (n = 6,697)	
	Mean	% of total	Mean	% of total	Mean	% of total
Burger	189 (9.8)	5.8 (0.3)	142 (12.1)†	4.9 (0.4)†	233 (12.3)	6.5 (0.4)
Pizza	142 (9.7)	4.4 (0.3)	102 (11.2)†	3.5 (0.4)^‡^	180 (15.4)	5.0 (0.4)
Sandwich	62 (4.8)	1.9 (0.2)	34 (4.4)†	1.2 (0.2)†	89 (8.8)	2.5 (0.2)
Chicken	42 (4.0)	1.3 (0.1)	37 (4.6)	1.3 (0.2)	48 (7.0)	1.3 (0.2)
Mexican	49 (4.9)	1.5 (0.1)	21 (5.1)†	0.7 (0.2)†	76 (8.0)	2.1 (0.2)
Asian	22 (3.5)	0.7 (0.1)	13 (3.5)^‡^	0.5 (0.1)^¶^	31 (5.7)	0.9 (0.2)
Coffee/snack	5 (1.1)	0.1 (0.0)	4 (1.5)	0.1 (0.1)	5 (1.5)	0.1 (0.0)
Fish	4 (1.4)	0.1 (0.0)	2 (0.8)	0.1 (0.0)	6 (2.5)	0.2 (0.1)
Other sources	2726 (33.9)	84.1 (0.5)	2548 (29.3)†	87.7 (0.7)†	2897 (51.7)	81.3 (0.7)
Total sodium	3242 (30.9)	–	2903 (26.7)†	–	3566 (49.8)	–

† p<0.001; ^‡^0.001<p<0.01; ^¶^0.01<p<0.05 for difference between two age groups. Significance testing conducted separately for mean and % of total sodium. Value in parentheses is the standard error.

**Table 4:** Mean energy from added sugars and % of total energy from added sugars (and standard errors) by FFR segment and age group.

	Total (n = 12,378)		Age 4–11 y (n = 5,681)		Age 12–19 y (n = 6,697)	
	Mean	% of total	Mean	% of total	Mean	% of total
Burger	21 (1.1)	6.2 (0.3)	17 (1.3)†	5.5 (0.4)^¶^	26 (1.3)	6.7 (0.3)
Pizza	3.4 (0.4)	1.0 (0.1)	3.0 (0.5)	1.0 (0.2)	3.9 (0.6)	1.0 (0.1)
Sandwich	3.3 (0.4)	1.0 (0.1)	1.7 (0.3)†	0.5 (0.1)†	4.9 (0.6)	1.3 (0.2)
Chicken	3.2 (0.4)	0.9 (0.1)	2.9 (0.6)	1.0 (0.2)	3.5 (0.6)	0.9 (0.2)
Mexican	3.0 (0.3)	0.9 (0.1)	1.3 (0.2)†	0.4 (0.1)†	4.6 (0.6)	1.2 (0.1)
Asian	0.5 (0.1)	0.2 (0.0)	0.2 (0.1)^¶^	0.1 (0.0)^¶^	0.8 (0.2)	0.2 (0.1)
Coffee/snack	0.8 (0.2)	0.2 (0.0)	0.8 (0.2)	0.3 (0.1)	0.8 (0.3)	0.2 (0.1)
Fish	0.3 (0.1)	0.1 (0.0)	0.2 (0.1)	0.1 (0.0)	0.4 (0.2)	0.1 (0.1)
Other sources	310 (4.0)	89.6 (0.4)	278 (4.8)†	91.2 (0.5)†	340 (5.8)	88.4 (0.4)
Total added sugars	346 (4.5)	–	305 (5.1)†	–	385 (6.4)	

† p<0.001; ^¶^0.01<p<0.05 for difference between two age groups. Significance testing conducted separately for mean and % of total added sugars. Value in parentheses is the standard error.

**Table 5:** Mean energy from solid fats and % of total energy from solid fats (and standard errors) by FFR segment and age group.

	Total (n = 12,378)		Age 4–11 y (n = 5,681)		Age 12–19 y (n = 6,697)	
	Mean	% of total	Mean	% of total	Mean	% of total
Burger	32 (1.7)	7.6 (0.4)	25 (2.1)†	6.4 (0.5)†	38 (1.9)	8.6 (0.5)
Pizza	20 (1.6)	4.8 (0.4)	15 (2.1)†	3.8 (0.5)^‡^	25 (2.3)	5.7 (0.5)
Sandwich	6.5 (0.5)	1.6 (0.1)	3.8 (0.5)†	1.0 (0.1)†	9.2 (0.8)	2.1 (0.2)
Chicken	6.1 (0.6)	1.5 (0.1)	5.7 (0.7)	1.4 (0.2)	6.6 (0.9)	1.5 (0.2)
Mexican	7.2 (0.7)	1.7 (0.2)	3.2 (0.8)†	0.8 (0.2)†	11.1 (1.2)	2.5 (0.3)
Asian	1.3 (0.3)	0.3 (0.1)	0.5 (0.1)^‡^	0.1 (0.0)^¶^	2.1 (0.6)	0.5 (0.1)
Coffee/snack	0.9 (0.2)	0.2 (0.0)	0.8 (0.2)	0.2 (0.1)	1.0 (0.2)	0.2 (0.1)
Fish	0.7 (0.3)	0.2 (0.1)	0.3 (0.1)	0.1 (0.0)	1.2 (0.5)	0.3 (0.1)
Other sources	344 (4.4)	82.1 (0.5)	339 (5.3)	86.2 (0.8)†	349 (6.5)	78.6 (0.7)
Total energy from solid fats	419 (4.0)	–	393 (5.3)†	–	443 (6.5)	–

† p<0.001; ^‡^0.001<p<0.01; ^¶^0.01<p<0.05 for difference between two age groups. Significance testing conducted separately for mean and % of total solid fats. Value in parentheses is the standard error.

**Figure 1:**
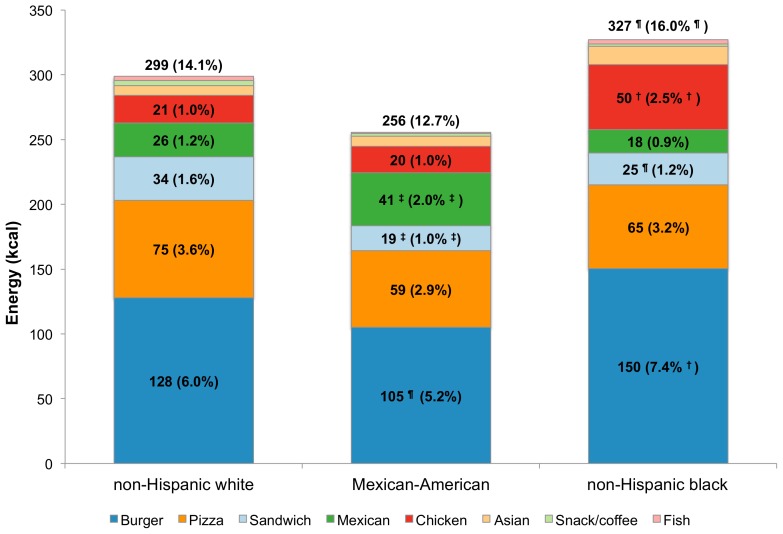
Estimated energy (kcal) intake and population proportion by FFR segment and race/ethnicity, age 4–19 y. ^†^p<0.001; ^‡^0.001<p<0.01; ^¶^0.01<p<0.05 for difference, with non-Hispanic whites as the reference group. Significance testing conducted separately for population mean and population proportion (value in parentheses is the population proportion).

**Figure 2:**
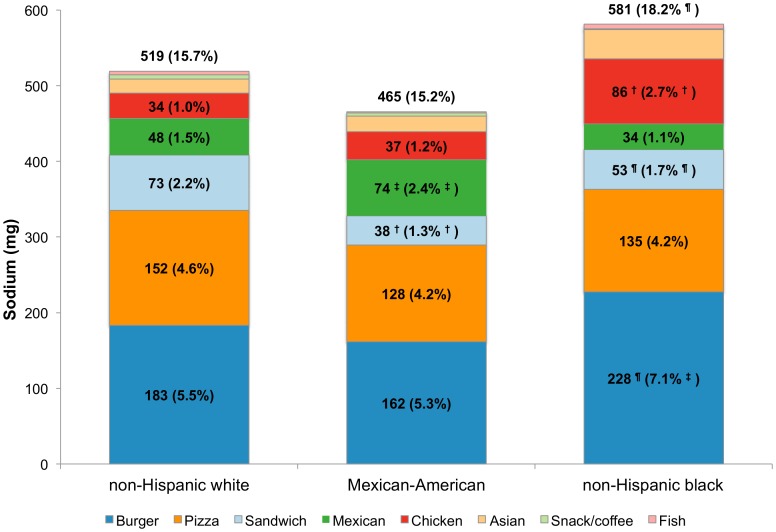
Estimated sodium (mg) intake and population proportion by FFR segment and race/ethnicity, age 4–19 y. ^†^p<0.001; ^‡^0.001<p<0.01; ^¶^0.01<p<0.05 for difference, with non-Hispanic whites as the reference group. Significance testing conducted separately for population mean and population proportion (value in parentheses is the population proportion).

**Figure 1** shows total energy intakes analyzed by fast food restaurant type and race/ethnicity. The distribution of energy intakes by FFR segment varied by race/ethnicity. Non-Hispanic black children derived more energy from fast food restaurants (327 kcal and 16.0% of total) than did non-Hispanic white children (299 kcal and 14.1% of total). Compared to non-Hispanic white children (6.0%), Mexican-American children derived significantly less energy from burger restaurants, whereas non-Hispanic black children derived significantly more (7.4%). By contrast, non-Hispanic white children derived significantly more energy from sandwich restaurants compared to the other race/ethnicity groups, while Mexican-American children derived more calories from Mexican restaurants when compared to non-Hispanic white children. Non-Hispanic black children derived significantly more energy from chicken restaurants than did non-Hispanic white children. From sandwich, Mexican, and chicken FFRs combined, there were no differences in energy by race/ethnicity (81 kcal for non-Hispanic white children, 80 kcal for Mexican-American children and 93 kcal for non-Hispanic black children). Comparable findings were obtained for the other dietary components (see **Figures 2** **–** **4** for sodium, energy from added sugars and energy from solid fats, respectively).

**Figure 3:**
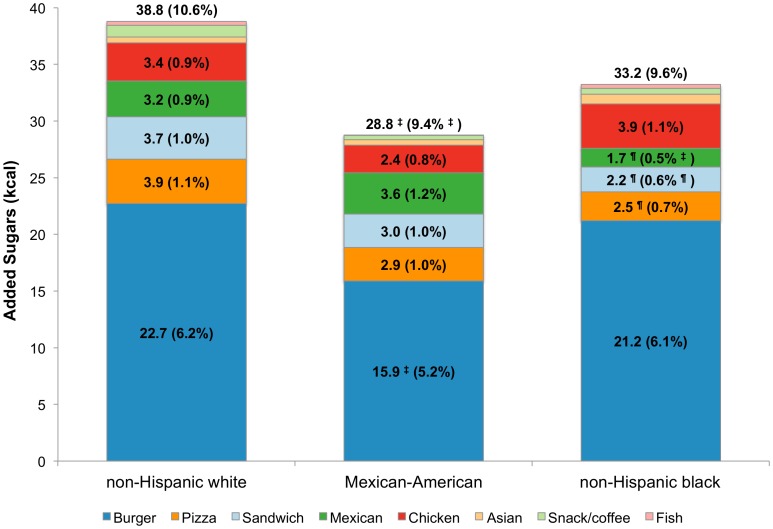
Estimated intake of energy from added sugars (kcal) and population proportion by FFR segment and race/ethnicity, age 4–19 y. ^†^p<0.001; ^‡^0.001<p<0.01; ^¶^0.01<p<0.05 for difference, with non-Hispanic whites as the reference group. Significance testing conducted separately for population mean and population proportion (value in parentheses is the population proportion).

**Figure 4:**
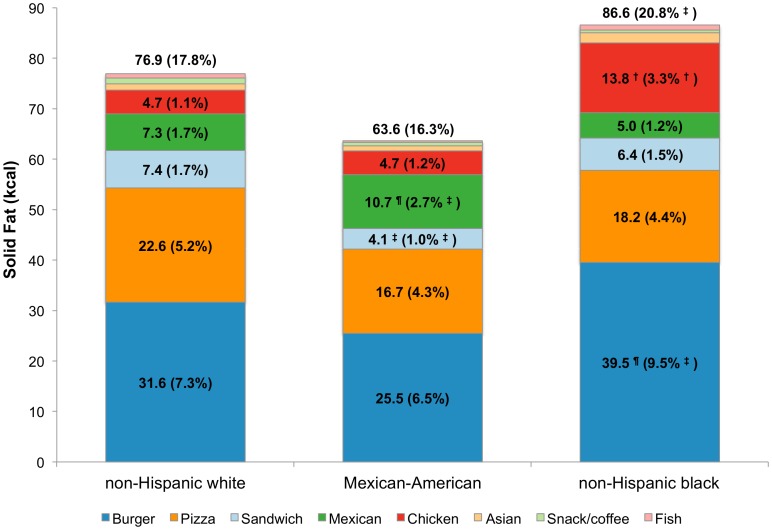
Estimated intake of energy from solid fats (kcal) and population proportion by FFR segment and race/ethnicity, age 4–19 y. ^†^p<0.001; ^‡^0.001<p<0.01; ^¶^0.01<p<0.05 for difference, with non-Hispanic whites as the reference group. Significance testing conducted separately for population mean and population proportion (value in parentheses is the population proportion).

### Energy-adjusted consumption of sodium, added sugars and solid fats by fast food restaurant type

**Table 6** shows the energy-adjusted amounts of sodium and percent of total energy from added sugars and solid fats by FFR type. The data were expressed as a percent of total energy, for solid fats and added sugars, or as mg/1,000 kcal for sodium. Overall, fast food restaurants contributed significantly more sodium per 1,000 kcal and percent of energy from solid fats than stores, while stores contributed significantly more energy from added sugars than FFRs. Pizza, sandwich, Mexican and Asian FFRs contributed significantly more sodium per 1,000 kcal than the burger segment. By contrast, pizza, sandwich, chicken, Mexican and Asian FFRs accounted for a lower proportion of their total energy from added sugars, when compared to burger restaurants. For percent of energy from solid fats, pizza FFRs contributed a greater proportion of total energy from solid fats than did burger FFRs, while Asian FFRs contributed the lowest proportion of total energy from solid fats.

**Table 6:** Mean sodium per 1,000(and standard errors) by FFR segment.

	Sodium		Added sugars		Solid fats	
	Sodium (mg) per 1,000 kcal	% of total sodium	% of total energy from added sugars^1^	% of total added sugars	% of total energy from solid fats^1^	% of total solid fats
FFR segment						
Burger (ref)	1458 (24)	5.8 (0.3)	16.5 (0.5)	6.2 (0.3)	25.3 (0.5)	7.6 (0.4)
Pizza	2028 (33)¶	4.4 (0.3)	4.5 (0.6)¶	1.0 (0.1)	30.6 (1.3)¶	4.8 (0.4)
Sandwich	2005 (76)¶	1.9 (0.2)	12.1 (0.9)¶	1.0 (0.1)	22.6 (0.9)	1.6 (0.1)
Chicken	1577 (67)	1.3 (0.1)	13.0 (1.1)¶	0.9 (0.1)	23.9 (0.8)	1.5 (0.1)
Mexican	1841 (36)¶	1.5 (0.1)	11.5 (0.9)¶	0.9 (0.1)	27.0 (0.8)	1.7 (0.2)
Asian	2590 (204)¶	0.7 (0.1)	6.2 (1.2)¶	0.2 (0.0)	16.6 (2.5)¶	0.3 (0.1)
Coffee/snack	1579 (267)	0.1 (0.0)	22.2 (4.4)	0.2 (0.0)	26.2 (1.8)	0.2 (0.0)
Fish	1420 (97)	0.1 (0.0)	14.3 (4.2)	0.1 (0.0)	28.8 (1.5)	0.2 (0.1)
Location of origin						
All FFR (ref)	1756 (20)	15.9 (0.5)	12.0 (0.4)	10.4 (0.4)	26.3 (0.4)	17.9 (0.5)
Store	1466 (11)¶	61.9 (0.7)	18.3 (0.3)¶	68.9 (0.6)	18.2 (0.1)¶	60.1 (0.7)
FSR	1892 (61)	7.0 (0.5)	13.8 (0.8)	4.5 (0.3)	23.1 (0.7)¶	6.2 (0.4)
School	1635 (78)	7.6 (0.5)	11.5 (0.5)	4.9 (0.4)	23.1 (0.5)¶	8.1 (0.5)
Other	1499 (40)¶	7.6 (0.3)	20.6 (0.9)¶	11.4 (0.5)	19.9 (0.7)¶	7.7 (0.4)

1 Value can be interpreted as the proportion of energy from each segment that is from added sugars or solid fats. For example, of all energy from burger FFRs, 16.5% came from added sugars.

¶ Indicates p-value <0.05. Reference group for segment analysis is burger. Reference group for location of origin is all FFR. Hypothesis testing only conducted for sodium (mg) per 1,000 kcal, and percent of total energy from added sugars and solid fats. Value in parentheses is the standard error.

## Discussion

The present analyses are the first example of stratifying consumption data for a nationally representative sample of US children by fast food restaurant type. First, analyses of consumption data are a useful supplement to the numerous prior analyses of fast food menu offerings [12]–[14]. Second, separating the FFR category into specific market segments as defined by the restaurant industry allows interested stakeholders to monitor the impact of public health policies and programs aimed at improving the quality of children’s diets.

The present analyses complement published studies of US diets by NHANES location of origin, where foods were aggregated based on a National Cancer Institute coding scheme [16], [17]. The present analyses are unique in that they accounted for all foods and beverages eaten at different types of fast food restaurants. Items consumed at burger restaurants may include burgers but may also include fries, chicken, salad, beverages, milkshakes, desserts, fruit and coffee. Thus, burgers alone contributed 1.2% of energy to the diets of the 6–11 y age group and 1.9% of energy to the 12–19 y age group, as reported previously [16]. By contrast, the total contribution of calories from burger restaurants was much higher: 6.2% overall, 5.4% for 4–11 y olds, and 6.8% for 12–19 y olds.

The present estimates, based on nationally representative federal datasets, were generally consistent with FFR segment sales, as published by industry sources [15]. Among FFRs, burger restaurants are the leading segment, as reflected in the consumption data. In 2009, total sales for burgers among leading national chains were $66 billion (or 47.7% of all FFR sales). The present estimate was that 43.5% (95% CI 40.2–47.4%) of FFR calories consumed by US children came from the burger segment. In this age group, pizza was far and away the second most important source of energy. Nationally, the sandwich, snack, chicken, pizza and Mexican segments were the 2^nd^–6^th^ leading segments in terms of sales, respectively. The infrequent consumption of coffee among children/adolescents explains why the snack/coffee segment was not observed as an important source of energy in this study. The present approach allows publicly available federal data to be used for identifying the relative importance of different FFR segments for specific demographic groups of interest, a definite advantage over the use of menu offerings or sales data. This approach can also be used to monitor population-based trends in dietary intakes from different types of FFRs.

The present consumption-based analyses complement past studies on the nutrient density of foods/meals from FFRs that are generally based on menu offerings [12]–[14], [28]–[31]. No nationally representative data on food consumption by FFR segment has previously been published. Merging market segmentation and public health surveillance approaches, the present method has the potential to transform ways in which the contribution of different food sources to the total diet can be evaluated and monitored over time. In particular, given efforts by the food and restaurant industry to improve the composition of menus and food products, a surveillance system needs to be established. The approach used here can also be adapted for a large number of dietary outcomes, including fruit, vegetable, low-fat dairy, fiber, potassium or any other dietary constituent or food group of interest.

The present study had several limitations. First, the present analyses were based on a 24-hour recall, which may result in under-reporting of foods perceived to be less healthful [32], [33]. This may result in a falsely minimized estimation of energy from both full-service and fast food restaurants or from food groups such as desserts, salty snacks, pizza or soda. However, such underreporting should not affect the relative rankings of consumption patterns by FFR segment. For younger children, dietary reporting by proxy respondent may result in under-reporting of foods consumed while the parent is not present. However, such a reporting error is less likely for restaurant foods, where parents are likely to be present, as opposed to school/childcare settings.

The NHANES food location of origin may also have some error. In particular, stores include supermarkets, grocery stores, convenience stores and pharmacies where people buy food. The FFR-segment coding algorithm also had some limitations; a small proportion of eating occasions could not be unambiguously assigned to a specific FFR category and were coded instead based on the relative market share of each segment.

Despite these limitations, the present work represents the only assessment of the contribution of different types of fast food restaurants to the diets of US children. The present analyses advance the field in two important ways. First, consumption data can supplement the ongoing analyses of menu offerings, while offering better insight into what is eaten. Second, intakes of energy and dietary constituents of public health concern can now be assigned to different types of fast food restaurants: burger, pizza, sandwich, chicken, Mexican, Asian, fish, and coffee/snack. The present approach can be usefully applied to monitor the effectiveness of industry and public health policies aimed at improving the dietary habits of American children.
